# Mitochondrial Dynamics in Type 2 Diabetes and Cancer

**DOI:** 10.3389/fendo.2018.00211

**Published:** 2018-04-27

**Authors:** Michelle Williams, M. Cecilia Caino

**Affiliations:** Department of Pharmacology, University of Colorado School of Medicine, Aurora, CO, United States

**Keywords:** mitochondria, fission, fusion, diabetes, cancer

## Abstract

Mitochondria are bioenergetic, biosynthetic, and signaling organelles that control various aspects of cellular and organism homeostasis. Quality control mechanisms are in place to ensure maximal mitochondrial function and metabolic homeostasis at the cellular level. Dysregulation of these pathways is a common theme in human disease. In this mini-review, we discuss how alterations of the mitochondrial network influences mitochondrial function, focusing on the molecular regulators of mitochondrial dynamics (organelle’s shape and localization). We highlight similarities and critical differences in the mitochondrial network of cancer and type 2 diabetes, which may be relevant for treatment of these diseases.

## Introduction

All living organisms rely on cellular and physiological mechanisms of homeostasis in order to maintain an internal environment optimal for life and function. Mitochondria are the foundation of cellular homeostasis, *via* their multiple roles in energy production, biosynthesis, calcium regulation and signaling, redox balance, and generation of reactive oxygen species. Not surprisingly, cells have evolved multiple mechanisms of quality control to ensure that mitochondria function at their best. These include protein import (1), folding and degradation (2), antioxidant defense mechanisms (3), mitochondrial turnover *via* autophagy (4), mitochondrial biogenesis (5), mitochondrial shape changes and cristae remodeling (6), and communication with the nucleus to coordinate transcriptional responses (7).

Emerging evidence indicate that mitochondrial dysfunction is associated with disparate diseases, including aging (8), neurodegenerative diseases (9), mitochondrial diseases (10), obesity (11), diabetes, and cancer. Although some controversies remain regarding whether functional or dysfunctional mitochondria are responsible for metabolic disorders, there is a resurgence of interest in understanding the mechanisms responsible for such mitochondrial alterations in disease. This review focuses on the molecular regulators of mitochondrial dynamics (organelle’s shape and localization) in cancer and metabolic pathologies.

## Regulation of Mitochondrial Dynamics

Mitochondria constantly undergo shape and number changes thanks to the two opposing processes of fission and fusion (12). In turn, changes in gross mitochondrial morphology and the interconnectivity of the mitochondrial network impact on energy production (13), calcium signaling, mitochondrial DNA distribution, apoptosis, mitophagy, and segregation of mitochondria between daughter cells (6). The fine-tuning of the fusion–fission balance is crucial for cellular fitness in response to extracellular stimuli and environmental stress (14). Thus, alterations of the fission–fusion balance lead to oxidative stress, mitochondrial dysfunction, and metabolic alterations.

At the molecular level, dynamin-like GTPases orchestrate mitochondria shape changes. The fission protein dynamin-related protein 1 (DRP1) assembles into ring-like structures to constrict mitochondrial membranes in a GTP-dependent manner (6). DRP1 is recruited to mitochondria by fission protein 1 (FIS1), mitochondrial fission factor (MFF), and the mitochondrial dynamic proteins of 49 (MiD49) and 51 kDa (MiD51). On the other hand, the fusogenic proteins mitofusin 1 and 2 (MFN1/2) are located in the outer mitochondrial membrane, and tether two mitochondria through homo- and hetero-typic dimerization (13). A single GTPase, optic atrophy protein 1 (OPA1), achieves fusion of the IMM.

An expanding number of degenerative disorders are associated with mutations in the genes encoding MFN2 and OPA1, including Charcot–Marie–Tooth disease type 2A and autosomal dominant optic atrophy (15). Defective mitochondrial dynamics seem to play a more general role in the molecular and cellular pathogenesis of common neurodegenerative diseases (Alzheimer’s and Parkinson’s) (14), as well as in cardiovascular disease (16), type 2 diabetes (T2D), and cancer.

## Mitochondrial Dynamics in T2D

**Figure 1:**
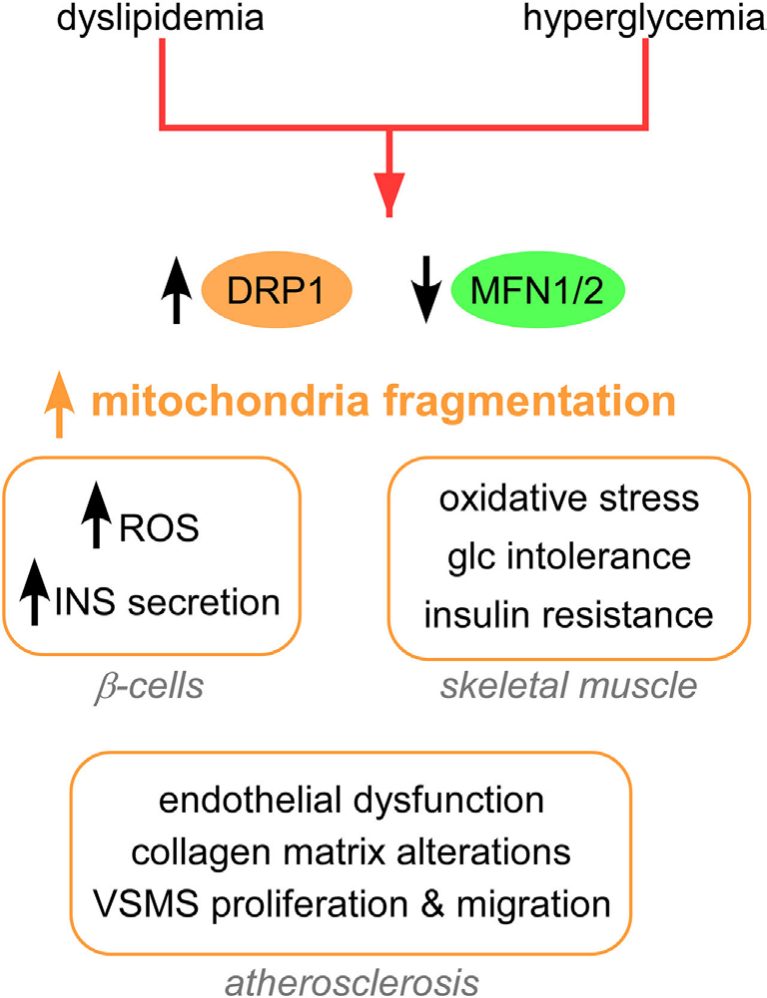
Mitochondrial shape alterations in T2D. Mitochondrial fragmentation and impaired mitochondrial trafficking are a hallmark of T2D. These changes in mitochondrial dynamics lead to pathological responses in β-cells, skeletal muscle, adipocytes, and vessels. Abbreviations: INS, insulin; Glc, glucose; T2D, type 2 diabetes.

The clinical complications of T2D include dyslipidemia, hyperglycemia (17), insulin resistance, and defects in insulin secretion from pancreatic beta cells (18). A major cause of such clinical complications is the increased production of mitochondrial ROS by hyperglycemia (17, 19). A common feature of mitochondrial morphology in T2D is an increased fragmentation (Figure 1), achieved *via* activation/upregulation of DRP1 and/or downregulation of MFN2 levels. In turn, increased fission and fragmentation of mitochondria was linked to HG-induced overproduction of ROS (20) and insulin secretion in mouse and human islets (21). Importantly, both HG-induced ROS and insulin secretion were blocked by inhibiting DRP1-induced fission. Furthermore, impaired mitochondrial fusion has been associated with insulin resistance in skeletal muscle (22) and with glucose intolerance and enhanced hepatic gluconeogenesis in a liver-specific MFN2 knockout (KO) mice (23). Interestingly, MFN2 KO led to increased ROS production, activation of JNK and endoplasmic reticulum (ER) stress response. Studies in rat models show that MFN2 overexpression improved insulin sensitivity and reduced lipid intermediates in muscle (24) and liver (25). At the molecular level, liver expression of MFN2 was associated with increased expression of the insulin receptor and the glucose transporter GLUT2, and activation of the PI3K/AKT2 pathway.

In addition, dyslipidemia models of T2D show increased mitochondrial fission (Figure 1). Excess palmitate (PA)-induced mitochondrial fragmentation and increased mitochondrion-associated DRP1 and FIS1 in differentiated muscle cells (26). In addition, PA induced mitochondrial depolarization, lower ATP synthesis and increased oxidative stress, and reduced insulin-stimulated glucose uptake (Figure 1). Both genetic and pharmacological inhibition of DRP1 attenuated PA-induced mitochondrial fragmentation and insulin resistance. In another study, DRP1 was induced in rat islets after stimulation by free fatty acids (FFAs), and this DRP-1 upregulation was accompanied by increased pancreatic β cell apoptosis (27).

Mitochondrial fission is associated with various processes that contribute to atherosclerosis in T2D (Figure 1), including endothelial dysfunction (28), collagen matrix alteration (29), and motility and proliferation of vascular smooth muscle cells (30). From a therapeutic standpoint, silencing FIS1 or DRP1 in venous endothelial cells isolated from patients with T2D blunted HG-induced mitochondrial fission and ROS production (28). Furthermore, metformin attenuated the development of atherosclerosis in diabetic mice by reducing DRP1-mediated mitochondrial fission in an AMP-activated protein kinase (AMPK)-dependent manner (31). Mitochondrial fission induced by DRP1 also plays a critical role in the pathogenesis of microvascular [nephropathy (32), retinopathy (33), and neuropathy] and macrovascular [stroke and myocardial ischemia (34)] complications of diabetes.

In summary, we know that many of the clinical complications of T2D are associated with mitochondrial fragmentation. We also know that tipping the balance toward increased mitochondrial fragmentation in mice leads to models of T2D. Furthermore, blocking DRP1 (or increasing MFNs) ameliorated hyperglycemia, dyslipidemia, and atherosclerosis in T2D models. Less clear are the mechanisms of alterations in expression and/or activity of DRP1/MFNs. Up to date, most of the studies have shown correlation between the hallmarks of T2D and increased fragmentation of mitochondria (Table 1). However, more studies should focus on understanding the spatiotemporal regulation of DRP1 and MFN1/2 levels during the natural progression of T2D. In this context, there are a number of open questions. For example, are there alterations on the regulation of DRP1/MFNs at the transcriptional, translational, or posttranslational level? Are DRP1/MFNs regulated by insulin, glucose, FFA signaling pathways? What are the tissue- and cell-specific differences in the regulation of mitochondrial shape in T2D? Identifying such molecular pathways controlling DRP1/MFN alterations in T2D might enable therapeutic efforts in prediabetic patients to prevent full-blown settlement of the disease.

**Table 1:** Mitochondrial dynamics in T2D and cancer.

Disease	Regulatory event	Molecular pathway	Cell function	Reference
T2D	DRP1 enrichment in calcified human carotid arteries	DRP1 controls matrix mineralization, cytoskeletal rearrangement, mitochondrial dysfunction, and reduced type 1 collagen secretion and alkaline phosphatase activity	Extracellular matrix changes in cardiovascular complications	(29)
	FFA	DRP1 leads to cytC release, caspase-3 activation, and generation of ROS	Apoptosis	(27)
	Hyperglycemia	ROCK1 phosphorylates DRP1	Nepropathy	(32)
	PA	Fragmentation was associated with increased oxidative stress, mitochondrial depolarization, loss of ATP production, and reduced insulin-stimulated glucose uptake	Insulin stimulated glucose uptake in skeletal muscle	(26)
	FIS1 and DRP1 increased in T2D patients	DRP1 induced ROS, and nitric oxide synthase activation	Endothelial dysfunction	(28)
	Hyperglycemia	HG leads to DRP1-mediated fragmentation and ROS	Cellular respiration	(20)
	Inflammatory signaling (TNF-α)	TNF-α induced MiR-106b which led to MFN2 downregulation	Insulin resistance	(23)
	Insulin	Unknown	Unknown	(30)
	Dyslipidemia	MFN2 prevents accumulation of lipid intermediates, including diacylglycerol and ceramides	Insulin resistance in skeletal muscle	(24)
	Dyslipidemia	MFN2 promotes the insulin signaling pathway (INSR/IRS2/GLUT2PI3K/AKT)	Insulin resistance in liver	(25)
	Hyperglycemia	MFN2 deficiency impaired insulin signaling in muscle and liver, induced ER stress, ROS production, and JNK activation	Insulin and glucose homeostasis	(23)

Cancer	Oncogenic MAPK signaling	RasG12V or BRAF^V600E^ activate ERK1/2, which then phosphorylates and activates DRP1	Mitochondria function and cell survival	(56)
	mTOR	mTORC1/4E-BP-dependent translation of MTFP1 leads to activation and recruitment of DRP1 to mitochondria	Cell survival	(58)
	Nestin	Nestin binds DRP1 and enhances DRP1 recruitment	Proliferation and invasion	(59)
	EHD1	EHD1 and Rabankyrin-5 interact with the retromer complex and induce VPS35-mediated removal of inactive DRP1 from mitochondrial membranes	Unknown	(60)
	AMPK	AMPK phosphorylates MFF, which increases DRP1 recruitment to mitochondria	Unknown	(61)
	SPOP loss-of-function mutants	SPOP mutations allow localization of INF2 to mitochondria, where it recruits DRP1	Cell migration and invasion	(62)
	SIRT4	SIRT4 inhibited Drp1 phosphorylation and weakened Drp1 recruitment to the mitochondrial membrane *via* an interaction with FIS1	Cell migration and invasion	(63)
	Estradiol	Estradiol stimulates mitochondria fission by decreasing MFN1/2 levels	Cell migration and proliferation	(66)
	Androgen	Androgens increase DRP1 expression *via* the AR	Cell proliferation	(65)

*The upstream regulators of mitochondrial shape are presented along with the molecular mechanisms at play*.

*SPOP, speckle-type POZ protein; FFA, free fatty acid; FIS1, fission protein 1; DRP1, dynamin-related protein 1; AMPK, AMP-activated protein kinase; MFF, mitochondrial fission factor; T2D, type 2 diabetes; ER, endoplasmic reticulum; MFN1/2, fusogenic proteins mitofusin 1 and 2; AR, androgen receptor*.

Another question that warrants further investigation is whether genetic susceptibility variants of DRP1 or MFNs are associated with T2D. A recent study in type 1 diabetes patients identified genetic factors associated with kidney disease (35). We propose that a similar approach in T2D patients could address to what extent genomic alterations of the mitochondrial shape genes are associated with disease. A potential association between genomic alterations of mitochondrial shaping genes and T2D might allow for better screening of susceptibility and/or risk prediction of certain T2D complications.

## Mitochondrial Dynamics in Cancer

Recent evidence indicates that mitochondrial shape, size, and localization regulate several of the hallmarks of cancer. For instance, mitochondrial shape dynamics have been linked to metabolic adaptation, cell cycle progression (36), necroptosis (19), apoptosis (37–39), autophagy (40), tumor growth, tumor cell motility (41, 42), invasiveness, and metastasis (43). The role of mitochondrial shape changes as regulators of cancer biology is reviewed in Ref. (44). Here, we will discuss recent insights into how mitochondrial dynamics are regulated in cancer.

When considering the common alterations in mitochondria shape, we find a dichotomy between tumors with enhanced mitochondrial fragmentation versus tumors with enhanced mitochondrial fusion. For instance, hepatocellular carcinoma (45), osteosarcoma (46), medulloblastoma (47), thyroid (42), colorectal (48), endometrial (49), and breast cancer (43) show increased mitochondrial fragmentation, due to upregulation of DRP1 levels and a concomitant reduction in MFN1/2 levels. On the other hand, tumors of the prostate (50), neuroblastoma (51), leukemia (52), glioblastoma (53), and lung (54) are associated with downregulation of DRP1 and increased MFN1/2 levels. What could be driving these contrasting preferences of fission versus fusion of the mitochondrial networks in cancer? Plausible explanations could lie on the genomic landscape, hormonal/growth factor context, tumor microenvironmental conditions, and therapy responses of the tumors in question.

Oncogenic and tumor suppressor signaling converge on mitochondria to reprogram cellular metabolism (55); thus, the particular genomic events driving a tumor might favor mitochondrial shape changes to meet the metabolic demands of the tumor cells. According to this hypothesis, oncogene-induced metabolic reprogramming should induce changes in mitochondrial shape. Indeed, recent studies show that oncogenic RasG12V, BRAF^V600E^ and MAPK/ERK (56, 57), mTOR (58) Nestin (59), and the endocytic protein EDH1 (60) increase DRP1-mediated mitochondrial fission. Similarly, the energy-sensing AMPK increased recruitment of DRP1 to mitochondria *via* phosphorylation of the MFF and (61). Speckle-type POZ protein loss-of-function mutations commonly found in primary prostate cancer were associated with increased DRP1 activation, mitochondrial fission, and prostate cancer cell invasion (62). Recently, loss of expression of the sirtuin SIRT4 was shown to lead to increased mitochondrial fragmentation (63). The signaling events that lead to DRP1 activation downstream of genomic and epigenetic alterations are summarized in Table 1.

In addition to the increasing number of oncogenes and tumor suppressors, growth factors and hormones regulate mitochondrial shape. Examples include Sonic Hedgehog (47), non-canonical Wnt ligands, pro-inflammatory cytokines, transforming growth factor-β, estradiol (64), and androgens (65). Estradiol promotes mitochondrial fragmentation through a reduction of MFN2 with parallel increase of FIS1 levels in ER+ breast cancer (66). From a translational standpoint, overexpression of MFN2 prevented estradiol-induced cell proliferation and motility (66). On the other hand, DRP1 is a transcriptional target of the androgen receptor, and androgen-stimulated DRP1 expression sensitizes prostate cancer cells to therapy-induced apoptosis (65). The possibility that other hormone-related malignancies exploit similar mechanisms of mitochondrial shape awaits further confirmation.

Tumor microenvironmental conditions exert yet another layer of regulation of mitochondrial shape. For instance, mitochondrial elongation is induced by nutrient deprivation in cancer cells (67). A hypoxic environment enhances mitochondrial fission in breast cancer (68) and glioblastoma (69). In this context, DRP1 was essential for hypoxia-stimulated cell motility. Indeed, silencing or expression of a dominant-negative mutant of DRP1 inhibited hypoxia-induced migration in both tumor cell models.

Finally, cancer cells also remodel their mitochondrial network in response to therapy. For instance, DRP1-mediated mitochondrial fragmentation is associated with cisplatin (68, 70), cytarabine and methotrexate (71), and tumor necrosis factor-related apoptosis-inducing ligand (TRAIL) (70) treatment among others. However, other therapeutic agents such as histone deacetylase inhibitors (72) produce the opposite effect, namely increased elongation of mitochondria. These opposite effects of therapy upon mitochondria morphology can be reconciled when considering the divergent signaling pathways elicited by the drugs. In the case of HDAC inhibitors, a decreased expression of FIS1 impaired DRP1 recruitment to mitochondria. These effects were independent of apoptosis induction. On the other hand, increased mitochondrial fragmentation on cisplatin and TRAIL-treated cells is coupled to apoptosis. Also worth considering, HDAC inhibitors could have additional roles in regulating mitochondrial morphology, due to non-histone-acetylating activity (acetylation of non-histone proteins, regulation of signaling kinases). A final consideration is the influence of the genomic background and tumor microenvironment on eliciting fission versus fusion upon therapy.

In summary, emerging evidence suggests that the contribution of the mitochondrial shaping genes to tumor cell biology is tumor type dependent and may reflect the genetic makeup, hormonal/growth factor context, tumor microenvironment conditions, and therapy responses of the tumor. Future efforts should aim to integrate these novel regulatory pathways and reach a comprehensive picture of the regulation of mitochondrial shape and function in cancer. Second, more emphasis should be directed toward identifying metabolic-dependent versus -independent functions of DRP1 and MFNs in cancer. For instance, which of the phenotypes associated with DRP1 activation in cancer are explained on basis of metabolism (increased glycolysis versus respiration)? Is it DRP1’s function on apoptosis (or mitochondrial localization) also important? A third area of interest for future research would be the development of anti-cancer therapies targeting mitochondrial dynamics. Encouraging fresh evidence indicates that modulating mitochondria morphology enhances anti-cancer therapies (73), particularly death receptor ligands (74–76) and antimitotic drugs (77).

## Targeting Mitochondrial Dynamics

The involvement of DRP1-mediated fission in disparate diseases settings has fueled the development of pharmacological approaches to inhibit mitochondrial fission. Mitochondrial division inhibitor-1 (mdivi-1) selectively impairs the GTPase activity of DRP1, without affecting the activity of dynamin-1, MFN1/2, or OPA1 (78). The mechanism of action of mdivi-1 involves allosteric binding and stabilization of a conformational form of unassembled DRP1 that cannot polymerize. mdivi-1 treatment induces rapid mitochondrial fusion, dampens ROS production and increases ATP production. Interestingly, the original report described a second function of DRP1 in mitochondrial outer membrane polarization (MOMP). DRP1 facilitated BAX/BAK-dependent MOMP in response to C8-BID or staurosporine, independently of mitochondrial fragmentation. Thus, mdivi-1 impaired staurosporine-induced apoptosis (78). Interestingly, mdivi-1 can induce apoptosis in DRP1-KO cells (79), suggesting that mdivi-1 has off-target effects. In contrast to these initial studies in which mdivi-1 prevented apoptosis, later studies showed that mdivi-1 sensitized cells to TRAIL-dependent apoptosis (74). This potentiation of apoptosis by mdivi-1 occurred through activation of mitochondrial and ER apoptosis pathways. Thus, these controversial results suggest that mdivi-1 can act either as pro- or anti-apoptotic pharmacologic agent, depending on the cell types and apoptotic stimuli in question (80).

In T2D models, mdivi-1 prevented mitochondrial fragmentation, oxidative stress and inflammation, and improved endothelial cell function (31). Another study showed that mdivi-1 prevented HG-stimulated insulin secretion in mouse and human islets (21). Furthermore, mdivi-1 rescued palmitate-induced mitochondrial dysfunction and ROS generation, as well as insulin resistance in skeletal muscle (26). Inhibition of Drp1 with mdivi-1 improved mitochondrial function and cardiac function in a model of myocardial ischemia/reperfusion of diabetic hearts (34).

In cancer cells, DRP1 inhibition has been shown to modulate therapy sensitivity, tumor metabolism, growth, and invasiveness. For instance, mdivi-1 suppressed mitochondrial autophagy, metabolic reprogramming, cancer cell viability, and motility of breast cancer cells (81). In regards to therapy modulation, mdivi-1 potentiated TRAIL-induced apoptosis in melanoma (74, 76) and ovarian cancer models (75). Furthermore, mdivi-1 induced cell death (75) and synergized apoptotic effects of platinum agents in drug resistant ovarian tumor cells (79). However, mdivi-1 prevents apoptosis induced by cisplatin in breast cancer (68) and leukemia (52). As discussed above, these controversial results suggest that mdivi-1 can act either as pro- or anti-apoptotic agent, depending on the cell types and apoptotic stimuli in question [reviewed in Ref. (80)]. Further investigations should address the precise mechanisms dictating the differential effects of mdivi-1 on cell survival.

Regarding the potential utility of mdivi-1 in the clinic, a number of questions remain open. For instance, what are the consequences of sustained *in vivo* inhibition of mitochondrial fission? What are the pharmacokinetics and cytotoxicity profiles for mdivi-1? Another point to consider is that mdivi-1 has poor solubility in water (80). This fact might limit the utility of mdivi-1 and might open the door for the design of new DRP1 inhibitors with improved solubility, specificity, and potency. In this regard, another pharmacological agent targets the recruitment of DRP1 to mitochondria *via* its interaction with FIS1. The small peptide inhibitor P110 blocks DRP1/FIS1 binding (82) and has shown promising results in neurodegenerative disease models. When tested in hepatocellular carcinoma, P110 blocked cell proliferation *in vitro* and *in vivo* (83). Future research will be needed to evaluate the utility of P100 both in T2D and cancer models.

## Conclusion

Given the metabolic alterations that are a hallmark of both T2D and cancer, it is not surprising that mitochondrial alterations are a shared feature in these disparate diseases. Over the past few years, we have learnt that mitochondria are not static, solitary organelles, but they rather undergo constant changes in morphology and subcellular distribution to meet the metabolic demands of the cell. Defects in mitochondrial dynamics play a role in the molecular and cellular pathogenesis of both T2D and cancer. Now, how similar or different are these two pathologies in regards to mitochondrial dynamics? In T2D, the literature unanimously reports an increase of mitochondrial fission mediated by DRP1. In cancer, most tumors follow this same pattern of increased DRP1-mediated mitochondrial fission. However, although less frequently, tumors might display augmented mitochondrial fusion *via* an increase of MFN1/2 levels and/or activity. How are these differences and similarities in the mitochondrial network explained at the molecular level? Up to date, most of the studies have shown correlation between T2D and altered mitochondrial shape. More studies should focus on understanding the spatiotemporal regulation of DRP1 and MFN1/2 levels and activity during the natural progression of T2D. Likewise, there is limited information on how the genetic, epigenetic, and microenvironmental factors influence mitochondrial dynamics, or which signaling pathways integrate extracellular stimuli with mitochondrial shape in T2D. Thus, due to this limited information, is not possible to conclude if T2D and cancer utilize similar or divergent mechanisms of control of mitochondrial shape. In this regard, it would be interesting to address how metabolic pathways commonly altered both in T2D and cancer impinge on mitochondrial morphology. Examples of such pathways include PI3K/AKT and AMPK. Another question that warrants further investigation is whether other aspects of mitochondrial biology are dysregulated in these diseases. For instance, are there alterations in mitochondrial quality control, mitochondria crosstalk to other organelles, or mitochondrial localization present in both T2D and cancer?

Regarding the use of DRP1 inhibitors as anti-T2D and -cancer agents, further studies should determine long-term effects of targeting mitochondrial dynamics *in vivo*, and establish the pharmacokinetics and cytotoxicity profiles for mdivi-1. In addition, the involvement of potential compensatory or resistance mechanisms to mdivi-1 has not been explored yet and should be addressed in the future. An area in need of further investment is the development of selective MFN1/2 inhibitors. Despite the existence of a few DRP1 inhibitors, there is no equivalent therapeutic agent to target fusion. The fact that several tumors show increased fusion might warrant further effort in this area.

## Author Contributions

MW performed literature search and review; MC conceived the project, designed the figures, and wrote the paper.

## Conflict of Interest Statement

The authors declare that the research was conducted in the absence of any commercial or financial relationships that could be construed as a potential conflict of interest.
